# Systemic inflammation in Fabry disease: a longitudinal immuno-genetic analysis based on variant stratification

**DOI:** 10.1177/26330040251375498

**Published:** 2025-09-11

**Authors:** Haylen Marín Gómez, Miguel López-Garrido

**Affiliations:** Internal Medicine, Hospital Universitario San Agustín de Linares, Área de Gestión Sanitaria Norte de Jaén – Servicio Andaluz de Salud (SSPA), Avenida San Cristóbal s/n, Linares, Jaén 23700, Spain; Cardiology, Hospital Universitario San Agustín de Linares, Área de Gestión Sanitaria Norte de Jaén – Servicio Andaluz de Salud (SSPA), Spain

**Keywords:** anti-ERT antibodies, complement system, Fabry disease, genotype-phenotype correlation, immune activation, inflammation

## Abstract

**Background:**

Fabry disease is a multisystemic lysosomal disorder caused by mutations in the GLA gene. Although traditionally attributed to lysosomal accumulation of globotriaosylceramide (Gb3), recent evidence suggests a key role of sustained systemic inflammation in its pathogenesis, even in early stages.

**Objectives:**

To characterize inflammatory and immunological profiles in a genetically stratified familial cohort with Fabry disease and explore genotype-dependent immune activation patterns.

**Design:**

Retrospective, longitudinal study of 11 patients from three interconnected families carrying distinct pathogenic GLA variants.

**Methods:**

We analyzed longitudinal data on inflammatory biomarkers (C-reactive protein, ferritin, fibrinogen) and immunological markers (IgG, IgM, IgE, complement C3/C4, anti-enzyme replacement therapy antibodies), alongside clinical variables. Multivariate correlation and unsupervised clustering techniques explored immunophenotypic patterns.

**Results:**

All patients exhibited chronic inflammation regardless of genotype. The c.53dup variant showed prominent humoral activation, IVS4+1G>A had complement-mediated activation with a cardiorenal phenotype, and c.845C>T showed mild persistent inflammation. Correlations included CRP and IgG, and complement factors with fibrinogen in the splicing variant group.

**Conclusion:**

Inflammation in Fabry disease is not merely a consequence of substrate accumulation but an active and early driver of disease. Preliminary inflammatory phenotypes based on immune mechanisms may guide future personalized therapeutic strategies.

## Plain language summary

Inflammation may help explain disease progression in Fabry even when organ damage is not yet visible

Fabry disease is a rare genetic condition where a specific enzyme does not work properly. This causes certain fatty substances to build up in the body’s cells over time, leading to damage in organs like the heart, kidneys, brain, and digestive system. Until now, it was believed that this buildup was the main cause of organ damage. However, our study suggests that the immune system may also play an important role—specifically, long-lasting inflammation. We analyzed 11 people from the same extended family with Fabry disease, each carrying a different genetic mutation. Alongside the usual clinical data, we examined their blood for markers of inflammation and immune activity. We found that all patients, regardless of the mutation they had, showed signs of chronic inflammation—even those with little or no visible organ damage. The pattern of immune activation differed depending on the mutation. Some patients had increased antibodies, others had overactive complement proteins, and some had mild but constant signs of inflammation. In a few cases, other immune-related diseases, such as autoimmune conditions, were also present. These findings suggest that inflammation may be a key part of Fabry disease from very early on, not just a result of damage. Recognizing this opens new possibilities for personalized treatment. While enzyme replacement therapy is the current standard, additional immune-based treatments might help patients whose disease keeps progressing despite standard care. In short, inflammation might be a silent driver of Fabry disease, and understanding its role could help improve outcomes, offer earlier interventions, and guide the development of more targeted therapies in the future.

## Introduction

Fabry disease is an X-linked lysosomal storage disorder caused by mutations in the *GLA* gene, leading to deficient activity of the enzyme α-galactosidase A. This enzymatic defect results in progressive accumulation of globotriaosylceramide (Gb3) and its deacetylated derivative lyso-Gb3, affecting multiple organ systems, particularly the heart, kidneys, nervous system, and vascular endothelium.^1,2^

Historically, lysosomal accumulation was considered the primary trigger of tissue damage. However, emerging hypotheses suggest that systemic inflammation plays an active role in disease progression, even in early stages or in patients with low substrate burden.^3–5^

This inflammatory axis is not exclusive to Fabry. Common conditions such as obesity, type 2 diabetes, and hypertension also share a chronic, low-grade inflammatory profile, where persistent elevation of cytokines like IL-1β, IL-6, and TNF-α acts as a catalyst for endothelial dysfunction, vascular injury, and disease advancement.^6,7^ A similar pattern has been described in Fabry disease, with immune activation preceding visible organ damage.^8–10^

Gb3, also known as CD77, functions as an immunologically active glycolipid capable of activating monocytes and dendritic cells, promoting the release of proinflammatory cytokines and generating a sustained inflammatory microenvironment.^11^ This activation may proceed through humoral or complement-mediated pathways, depending on the underlying genetic variant and clinical context. Additionally, the emergence of neutralizing or non-neutralizing anti-enzyme replacement therapy (anti-ERT) antibodies introduces further immunological complexity, with potential implications for disease severity and treatment response.^12–14^

Despite increasing evidence, inflammation remains an underappreciated component in Fabry pathogenesis. Prior studies have shown elevated levels of proinflammatory cytokines in plasma and tissues, even in children or females with minimal clinical expression.^15–17^ Endothelial dysfunction has been demonstrated histologically before structural injury is evident, supporting inflammation as an initiating event rather than a secondary process.^18–20^

In this context, incorporating inflammatory and immunological biomarkers into the routine follow-up of Fabry patients could provide essential information for early risk stratification and personalized management. This study aims to describe inflammatory profiles in a familial cohort harboring three distinct *GLA* variants, evaluating genotype-specific immune activation patterns and exploring their potential clinical relevance. We also propose an immunopathological framework to guide future therapeutic strategies beyond enzyme replacement.

## Methods

### Study design

This was a retrospective, observational, and longitudinal study conducted on a familial cohort of patients with genetically confirmed Fabry disease.

### Population

A total of eleven patients were included, all belonging to three interconnected family clusters. Each cluster carried one of three distinct pathogenic variants in the *GLA* gene:

- c.53dup; p.Leu19Profs*12 (nonsense mutation; *n* = 7)
- IVS4+1G>A (splicing mutation; *n* = 3)
- c.845C>T; p.Thr282Ile (missense mutation; *n* = 1)

Diagnosis was confirmed through molecular analysis and enzymatic activity assessment. All patients had at least 2 years of follow-up data and were evaluated under a standardized institutional protocol.

### Variables and data collection

Longitudinal data were extracted from clinical records, including:

- *Inflammatory biomarkers*: C-reactive protein (CRP), fibrinogen, and ferritin.
- *Immunological parameters*: Serum IgG, IgM, IgE, complement fractions (C3 and C4), and anti-ERT IgG antibodies (quantified by ELISA when available).
- *Markers of organ involvement*: Estimated glomerular filtration rate (eGFR), proteinuria (mg/day), NT-proBNP, and troponin T.
- *ERT status*: Type of enzyme replacement therapy (agalsidase alfa or beta), treatment duration, antibody status, and clinical tolerance.

Therapeutic evolution and relevant clinical events—including cardiovascular complications, renal progression, neurological symptoms, and autoimmune phenomena—were also recorded.

### Statistical analysis

For each patient, central tendency (mean, median) and dispersion (standard deviation, range) values were calculated and grouped by genetic variant. Correlation analysis was performed using the Spearman rank test to assess associations between inflammatory and immunological markers.

Additionally, principal component analysis (PCA) and hierarchical clustering (heatmaps) were conducted to explore immunophenotypic patterns and intra-group variability. All statistical analyses and visualizations were performed using Python 3.9, leveraging the pandas, scipy, numpy, seaborn, and matplotlib libraries. A *p*-value <0.05 was considered statistically significant.

This study was reported following the STROBE guidelines for observational studies (Supplemental Material).

## Results

### Clinical and immunological profiles by genetic variant

Baseline clinical, immunological, and genetic characteristics are presented separately for each genotype in Tables 1–3.

**Table 1.** Clinical features by patient for the genetic variant c.53dup; p.Leu19Profs*12 (nonsense mutation, *n* = 7).

Genetic variant 53dup; p.Leu19Profs*12.							
	Patient 1	Patient 2	Patient 3	Patient 4	Patient 5	Patient 6	Patient 7
Sex	Male	Male	Male	Female	Female	Female	Female
Clinical phenotype	Late onset	Late onset	Late onset	Late onset	Late onset	Late onset	Late onset
Diagnosis	2021 (Family history of index case)	2021 (Family history of index case)	2021 (Family history of index case)	2021 (Family history of index case)	2021 (Family history of index case)	2021 (Family history of index case)	2021 (Family history of index case)
Enzyme replacement therapy (ERT)	Agalsidase beta	Agalsidase alfa	Agalsidase alfa	Agalsidase alfa	Agalsidase alfa	Agalsidase beta	Agalsidase beta
ERT start date	2022	2022	2021	2021	2022	2021	2022
Age	51 years	34 years	21 years	62 years	59 years	30 years	17 years
Initial α-galactosidase A activity (Dry Blood Spot method)	Absent	Absent	Absent	1 μmol/L/h	2.9 μmol/L/h.	0,7 μmol/L/h	1 μmol/L/h
Lyso Gb3 (measured in DBS)	60 ng/mL	25 ng/mL	75 ng/mL	7.33 ng/mL	2.93 ng/mL	5.55 ng/mL	2.35 ng/mL
Ac Anti ERT	10689,78 μg/mL	Absent	171,64 μg/mL	188,75 μg/mL	Absent	Absent	Absent
Autoimmunity	Type 1 hypersensitivity (2022)	Autoimmune pancreatitis (2025)	Not documented	Not documented	Inflammatory bowel disease (2023–2024)	Not documented	ANCA PR3-positive vasculitis—Rapidly progressive glomerulonephritis in 2023
Relevant Clinical Features of Fabry Disease	→ Moderate proteinuria, no angiokeratomas, no corneal changes, denies sensory disturbances	Moderate proteinuria	Progressive renal failure requiring hemodialysis, coexisting with grade IV hydronephrosis linked to underlying disease.	Clinically established heart failure, significant proteinuria, and a massive ischemic stroke.	Sustained proteinuria (up to 266 mg/24 h), visceral inflammation associated with Fabry disease, and neuropathic pain.	Small vessel pathology on brain MRI, and acroparesthesia	Highly variable glomerular filtration rate (ranging from normal to 44 mL/min), fluctuating creatinine with post-inflammatory peaks, significant proteinuria (>400 mg/day), decline in GFR following ANCA PR3+ vasculitis event, and acroparesthesias.
Cardiac involvement	→ Severe hypertrophy, mild dysfunction, elevated biomarkers	Dilatation and hypertrabeculation, less functionally impaired	Midmyocardial late gadolinium enhancement in the basal inferolateral segment,	Severe left ventricular hypertrophy since at least 2014,	Symmetric concentric hypertrophy without fibrosis, subcortical chronic ischemia	Mild proteinuria, late enhancement on cardiac MRI	None
Inflammatory Findings	Markedly elevated IgM, positive anti-ERT antibodies	Moderate IgM, negative anti-ERT antibodies	Elevated immunoglobulins, with IgG 1857 mg/dL Persistently elevated CRP, mean 81.5 mg/L	Chronically elevated CRP (mean 36.4 mg/L) with peaks >60 mg/L, persistently elevated fibrinogen (mean >700 mg/dL), and elevated LDH indicating tissue damage.	Sustained elevation of CRP, fibrinogen, and fecal calprotectin, with no evident infectious pattern. Persistently elevated IgM and IgE, with no deficiency pattern.	IgG (1240 mg/dL) and CRP 3.6 mg/L No complement consumption	ANCA PR3-positive Complement consumption Sustained elevation of IgG and IgM over time
Inflammatory Status	Low-grade chronic inflammation	Sustained, even without immune response to ERT	Severe systemic inflammation	Chronic low-grade inflammatory state with flares	Markedly elevated systemic inflammation	Low-grade chronic inflammation	Multiorgan inflammation due to ANCA-associated vasculitis and immunosuppressive treatment.
Current Status	Alive—Stable	Alive—Stable	Hemodialysis. Awaiting kidney transplant	Deceased—Massive ischemic stroke (12/2023)	Alive—Stable	Alive—Stable	Proteinuria and chronic kidney disease (immune + Fabry )

**Table 2.** Clinical features by patient for the genetic variant IVS4+1G>A (*n* = 3).

Clinical characteristics	Patient 8	Patient 9	Patient 10
Sex	Female	Female	Male
Clinical phenotype	Late onset	Late onset	Late onset
Diagnosis	2000 Family history of index case	2000 Family history of index case	1998 Family history of index case
Enzyme replacement therapy (ERT)	Agalsidase alfa (2021) Agalsidase beta (2024)	Agalsidase alfa (2021)	Agalsidase alfa (2000)
ERT start date	2021	2021	2000
Age	63 years	59 years	60 years
Initial α-galactosidase A activity (Dry Blood Spot method)	1 μmol/L/h. (Measured prior to ERT)	3.5 μmol/L/h. (Measured prior to ERT)	Absent (Reported in 1998 report)
Lyso Gb3 (measured in DBS)	10 ng/mL	2.7 ng/mL	48 ng/mL
Ac Anti ERT	Negative	Negative	Negative
Autoimmunity	**Not documented**	**Not documented**	Partial graft rejection
Relevant Clinical Features of Fabry Disease	No follow-up between 2010–2021, but undertreatment was documented; Delayed diagnosis hindered timely intervention.	→ No evident clinical renal involvement → likely partial expression	Early renal transplant, persistent proteinuria, IgG antibodies against agalsidase (neutralizing antibodies not assessed).
Cardiac involvement	Clinical onset with chest pain → established and Severe LVH	Mild left ventricular hypertrophy	Severe LVH, biventricular dysfunction, late gadolinium enhancement, pulmonary hypertension → typical pattern of infiltrative cardiomyopathy.
Inflammatory Findings	→ Elevated LDH and D-dimer (endothelial damage) → High complement levels	More stable CRP levels, milder and slower disease progression	CRP >90, persistently elevated NLR, lymphopenia, high ferritin and fibrinogen → indicative of a chronic active inflammatory state.
Inflammatory Status	Mild-to-moderate inflammation, with intermittent flares	Practically asymptomatic	Chronic active inflammatory state
Current Status	→ Disease progression → Despite early diagnosis (through “carrier” screening), ERT was initiated late due to advanced clinical presentation.	→ Follow-up – Stable	→ Progressive deterioration, evidence of cardiac fibrosis. → Limited benefit from ERT

This table summarizes the clinical and demographic characteristics of patients carrying the IVS4+1G > A variant (*n* = 3), including sex, age at diagnosis, clinical phenotype, enzyme activity, biomarker levels, treatment, and immune response.

**Table 3.** Clinical features by patient for the genetic variant c.845C>T (*n* = 1).

Clinical characteristics	Patient 11
Sex	Female
Clinical phenotype	Late onset
Diagnosis	2000 Family history of index case
Enzyme replacement therapy (ERT)	Agalsidase beta (2024)
ERT start date	2024
Age	53
Initial α-galactosidase A activity (Dry Blood Spot method)	3 μmol/L/h. (Measured prior to ERT)
Lyso Gb3 (measured in DBS)	20 ng/mL
Ac Anti ERT	Negative
Autoimmunity	**Not documented**
Relevant clinical features of Fabry disease	Moderate proteinuria
Cardiac involvement	Yes (progressive LVH)
Inflammatory findings	Mildly elevated, Inflammatory markers (LDH, Ferritin)
Inflammatory status	Low-grade sustained inflammation
Current status	Alive – Stable

This table details the clinical and demographic characteristics of the single patient carrying the c.845C > T variant, including age at diagnosis, clinical presentation, enzyme activity, biomarker levels, treatment, and immune response.

**Figure 1.**
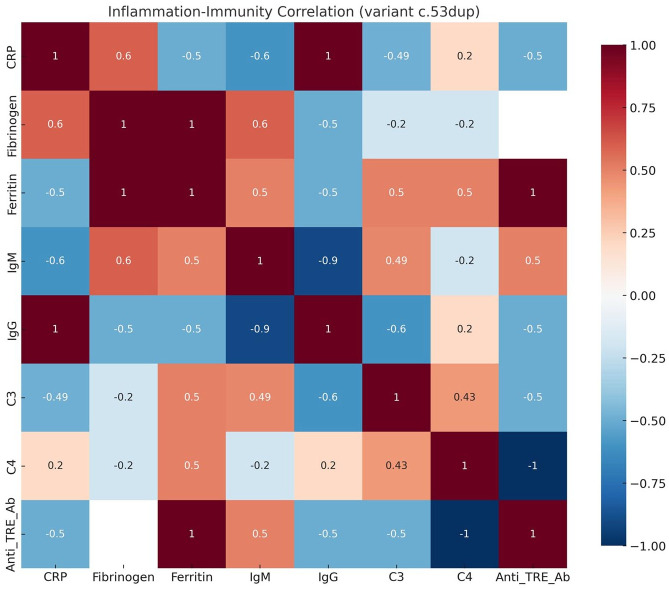
Inflammation–Immunity Correlation Matrix (variant c.53dup). Heatmap displaying pairwise correlation coefficients between inflammatory and immunological markers in patients with the c.53dup variant. The color gradient represents correlation strength and direction (–1 to +1).

**Figure 2.**
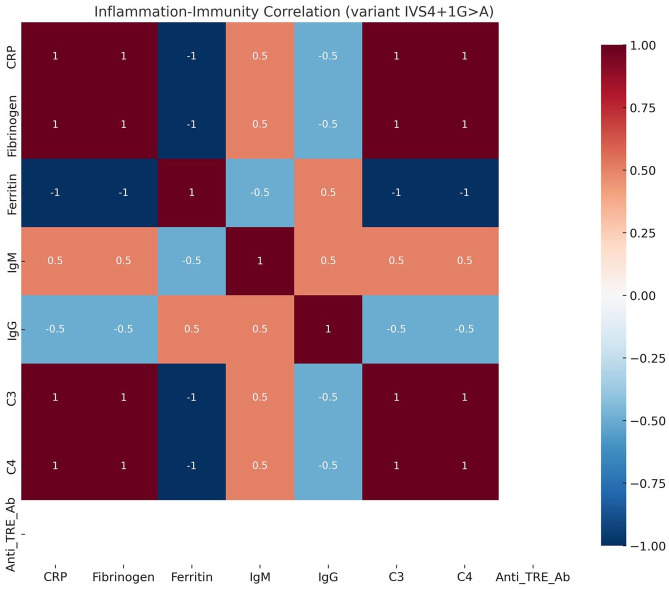
Inflammation–Immunity Correlation Matrix (variant IVS4+1G>A). Heatmap showing pairwise correlation coefficients between inflammatory and immunological markers in patients carrying the IVS4+1G>A variant. Color scale indicates the strength and direction of correlation (–1 to +1).

- *Table 1 (c.53dup; n = 7)*: Patients exhibited a multisystemic, cardiac-variant phenotype with variable severity and persistent systemic inflammation, including elevated immunoglobulins, complement fractions, and anti-ERT antibody titers. As shown in Figure 1.
- *Table 2 (IVS4+1G>A; n = 3)*: This subgroup showed a more stable cardiorenal phenotype with moderate-to-severe inflammatory activity, characterized by elevated complement fractions and fibrinogen levels suggesting complement-mediated inflammation. The correlation between inflammatory and immune markers is shown in Figure 2.
- *Table 3 (c.845C>T; n = 1)*: This patient had a cardiac-predominant phenotype with persistently elevated NT-proBNP and mild but sustained systemic inflammatory markers, despite absence of anti-ERT antibodies.

ERT type was selected based on availability and local prescribing practices. Lyso-Gb3 measurements were performed prior to initiation of ERT unless otherwise indicated.

## Discussion

Traditionally, Fabry disease has been conceptualized as a linear process where progressive accumulation of globotriaosylceramide (Gb3) and lyso-Gb3 leads to multiorgan damage, especially in kidneys, heart, and endothelium.^11,12^ However, this model fails to fully explain early or atypical manifestations, including those seen in females or patients with minimal substrate load.^13–15^

Recent evidence suggests that inflammation acts as a primary, genotype-modulated axis influencing disease expression from early stages.^16,17^ In our cohort, this was most notable in patients carrying the c.53dup variant, who showed strong humoral activation with elevated IgG, IgM, and anti-ERT antibodies. These immune patterns were associated with immune-mediated complications such as ANCA-positive vasculitis, suspected autoimmune pancreatitis, and inflammatory bowel disease.

Although some of these diagnoses lacked complete histological confirmation, their frequency and clustering within a single family suggest an underlying immune predisposition. Prior studies have reported associations between Fabry disease and autoimmune conditions like systemic lupus erythematosus, Crohn’s disease, and autoimmune thyroiditis.^18–20^ While causality remains unclear, chronic immune activation may favor secondary immune dysregulation.^21^

At the molecular level, Gb3 (CD77) can activate dendritic cells, monocytes, and vascular endothelium,^22^ promoting secretion of pro-inflammatory cytokines and sustaining a chronic inflammatory microenvironment. Importantly, these immune effects may precede irreversible tissue damage.^23^ Experimental models confirm early upregulation of TNF-α, IL-1β, and IL-6 before histological fibrosis or necrosis appears.^24–26^ Endothelial dysfunction, a hallmark of Fabry disease, is also detected early with oxidative stress and cytokine release even in the absence of significant Gb3 accumulation.^27–29^ Our findings align with these data, as patients with low lyso-Gb3 levels still exhibited elevated CRP, ferritin, or complement fractions.

Patients with the IVS4+1G>A variant demonstrated a complement-mediated phenotype, with consistent elevations in fibrinogen, C3, and C4 despite the absence of anti-ERT antibodies. This pattern may reflect lectin or classical pathway activation, as previously suggested in complement-focused studies.^30^ Anti-ERT antibodies are increasingly recognized not just as pharmacological inhibitors but as active immunological players capable of neutralizing enzymatic activity, promoting substrate accumulation, and inducing infusion reactions.^31–33^ In our cohort, IgM levels correlated with anti-ERT titers in the c.53dup subgroup, supporting B-cell-driven immunogenicity in Fabry pathophysiology.

Based on these findings, we propose a *preliminary classification* of inflammatory phenotypes in Fabry disease, intended as a *hypothesis-generating framework* for future studies:

- *Humoral phenotype:* Elevated IgG, IgM, and anti-ERT antibodies suggesting potential benefit from B-cell-targeted approaches.
- *Complement-mediated phenotype:* Elevations in C3/C4 and fibrinogen, supporting exploration of complement inhibitors.
- *Low-grade systemic phenotype:* Persistent mild CRP or ferritin elevation, possibly amenable to broader anti-inflammatory strategies.

This classification represents a shift from a purely substrate-centered model to a multiaxial immunopathological perspective that may inform personalized interventions in Fabry disease and other lysosomal storage disorders.^34^

Additionally, recent studies on complement-inhibitor therapies such as narsoplimab and iptacopan highlight the therapeutic potential of targeting complement pathways in Fabry disease, especially in patients exhibiting complement-mediated inflammatory profiles.^35^ These agents may provide a rationale for future clinical trials aimed at reducing immune-mediated organ damage. Furthermore, emerging reviews have proposed the investigation of immune checkpoint modulation in Fabry disease as a novel approach to regulate excessive immune activation, although this remains highly exploratory and warrants robust preclinical validation.^36^

Recent cardiac MRI studies reinforce this concept by showing myocardial edema as a precursor to fibrosis, suggesting inflammation as an early disease driver.^37^ Moreover, comparative imaging data indicate that patients receiving ERT or chaperone therapy experience reductions in T2 values—a surrogate of edema and inflammation—while untreated patients exhibit increases, supporting an anti-inflammatory role of these treatments.^38^

In clinical practice, monitoring biomarkers such as CRP, complement fractions, and immunoglobulin levels may allow earlier risk stratification and more informed treatment decisions. For patients with refractory or atypically progressive disease, adjunct immunomodulatory strategies—including low-dose corticosteroids, IL-6 inhibitors, or selective immunosuppressants—should be carefully explored in research settings or compassionate use contexts.^39–41^

Finally, the case of P7—a teenager with Fabry disease and biopsy-confirmed ANCA PR3-positive vasculitis—underscores the real-world impact of unrecognized immune activation. Although rare, such presentations echo previous reports of Fabry disease mimicking systemic vasculitides or coexisting with autoimmune syndromes>.^39,40^

In conclusion, inflammation in Fabry disease should not be viewed as a secondary phenomenon or late complication. It is a central and dynamic process interacting with genotype, enzymatic activity, and clinical phenotype—ultimately shaping prognosis and therapeutic response. Recognizing this complexity offers a pathway toward more personalized and effective management, advancing beyond enzyme replacement into the era of immunological precision medicine.

### Limitations

This study constitutes one of the most extensive immunological characterizations to date within a familial cohort affected by Fabry disease. Nonetheless, certain limitations must be acknowledged to ensure an objective appraisal of its findings.

First, the limited sample size reflects the inherent rarity of Fabry disease and the constraints of single-center cohorts. While this may reduce statistical power and limit generalizability, the inclusion of genetically and environmentally linked individuals within a defined lineage offers a unique internal consistency and allows controlled analysis of genotype–phenotype correlations—an approach seldom feasible in multicentric designs.

Second, although the study incorporated longitudinal monitoring of immunological and inflammatory biomarkers, detailed cytokine profiling and lymphocyte subpopulation analyses were not systematically performed. These parameters may provide deeper insight into the immunopathogenesis of Fabry disease, particularly with regard to dominant inflammatory phenotypes. Future studies should consider incorporating such markers—including TNF-α, IL-6, IL-1β, and B- and T-cell panels—to better delineate immune activation pathways.

Third, anti-ERT antibodies were quantified using validated immunoassays; however, the neutralizing potential of these antibodies was not assessed. Although functional interference remains speculative, the documented correlations between antibody titers, immunoglobulin levels, and clinical outcomes suggest a meaningful immunological role warranting further investigation.

Additionally, this was a single-center study conducted under a standardized institutional protocol. While this ensured consistency in data collection and interpretation, the findings require external validation through larger multicenter efforts. Such studies could account for interlaboratory variability and broaden the applicability of the proposed inflammatory stratification model.

Despite these limitations, the study provides robust and clinically relevant evidence supporting inflammation as a transversal and dynamic axis in Fabry disease. The integration of clinical, biochemical, and immunological parameters across multiple time points strengthens the internal validity of the observations. These results underscore the potential utility of immune-based stratification models to guide personalized therapeutic decisions in Fabry and, by extension, in other lysosomal disorders.

It is expected that these preliminary findings will stimulate future collaborative studies in specialized centers, where advanced immunophenotyping and molecular techniques may help consolidate the role of inflammation as both a biomarker and a therapeutic target in Fabry disease.

## Conclusion

This study supports a new interpretation of Fabry disease that goes beyond the traditional classical/nonclassical categorization, emphasizing inflammation as a shared, early, and active driver across different GLA variants.^16,21,24^ Our findings highlight that chronic immune activation is not simply a downstream effect of substrate accumulation but a central component of disease pathophysiology.

Recognizing inflammation as an early and modifiable axis may enable the development of more precise diagnostic, prognostic, and therapeutic approaches. The observed coexistence of autoimmune phenomena, such as ANCA PR3-positive vasculitis in P7, underscores the need for systematic immunological surveillance in Fabry patients, particularly those with suggestive personal or family histories^38,39^ Additionally, the correlation between anti-ERT antibodies and broader immune activation suggests their potential role as functional biomarkers rather than mere therapeutic obstacles.^26,33^

Routine assessment of immune-inflammatory biomarkers—including high-sensitivity CRP, complement fractions, immunoglobulin levels, and cytokine panels—may help identify patients at risk of progression and inform research into adjunctive immunomodulatory strategies.^22,24,35^

Looking ahead, clinical trials exploring the combination of enzyme replacement therapy with targeted immunomodulatory approaches represent a logical avenue for investigation, particularly for patients with suboptimal ERT responses or atypical clinical presentations not fully explained by substrate accumulation.

In summary, inflammation in Fabry disease should not be seen as a secondary or late complication but as a central and potentially modifiable axis of disease. Acknowledging this complexity may ultimately improve disease control and patient quality of life.^21,37^

## Supplemental Material

sj-doc-1-trd-10.1177_26330040251375498 – Supplemental material for Systemic inflammation in Fabry disease: a longitudinal immuno-genetic analysis based on variant stratification Supplemental material, sj-doc-1-trd-10.1177_26330040251375498 for Systemic inflammation in Fabry disease: a longitudinal immuno-genetic analysis based on variant stratification by Haylen Marín Gómez and Miguel López-Garrido in Therapeutic Advances in Rare Disease

## Declarations

**Ethics approval and consent to participate:** The study was approved by the Ethics Committee of Hospital Alto Guadalquivir (Approval Code: CEIm HAG 01/2022) and Hospital Universitario San Agustín de Linares (Approval Code: CEIm HUSA 02/2022). Informed consent for participation was obtained verbally and in writing from all patients or their legal representatives.

**Consent for publication:** No identifiable personal data, images, or videos were included in this manuscript. Therefore, consent for publication is not applicable.

**Availability of data and materials:** The data supporting the findings of this study are available from the corresponding author upon reasonable request.
